# ​Consideration of sex/gender aspects in cardiovascular clinical trials

**DOI:** 10.1007/s00392-025-02793-3

**Published:** 2025-11-26

**Authors:** Maximilian Bley, Linda Mathez, Susanne Menz, Isabella Stephan, Vera Regitz-Zagrosek, Marc J. Lerchenmueller, Carolin Lerchenmüller

**Affiliations:** 1 Chair for Gender Medicine, University of Zurich, Zurich, 8006 Switzerland; 2 Department of Cardiology, University Hospital Zurich, Zurich, 8091 Switzerland; 3 Institute of Gender in Medicine, Charité Universitaetsmedizin Berlin, Berlin, 10115 Germany; 4 University of Mannheim, Mannheim, 68161 Germany; 5 Leibniz Centre for European Economic Research, Mannheim, 68161 Germany; 6 Department of Cardiology, University Hospital Heidelberg, Heidelberg, 69120 Germany; 7 German Centre for Cardiovascular Research (DZHK), Partner Site Heidelberg/Mannheim, Heidelberg, 69120 Germany

**Keywords:** Gender equity, Clinical trials, Cardiovascular medicine, Participation, Reporting

## Abstract

**Background:**

Understanding sex/gender in the context of health and disease is critical to deliver the best care. However, sex/gender have not been consistently considered in cardiovascular clinical trials. Global initiatives, including the Sex and Gender Equity in Research (SAGER)-guidelines, aim to improve the quality of the reporting, but it remains unclear if they are used consistently.

**Methods:**

We conducted a systematic analysis of cardiovascular clinical trials published in PubMed between 2018 and 2024. To investigate the representation of women/females, we first analyzed the participation-prevalence-ratio (PPR). Second, we measured sex/gender-sensitive reporting (SGR) applying modified SAGER-guidelines. In addition, we determined whether study author sex/gender impacts the other variables.

**Findings:**

We identified 1593 clinical trials with a total of 716,569 woman/female participants (38.5%). The median PPR of all trials remained suboptimal at 0.77 (95%-CI: 0.74–0.79) throughout the years with a modest positive trend towards 2024 and significant underrepresentation in some disease entities (e.g., ischemic heart disease, heart failure). Analyzing an evenly distributed sample of 632 trials, we found suboptimal SGR, especially for endpoints and discussions. We found a positive correlation of increased participation of women/females and SGR with women/females as authors.

**Interpretation:**

Our results suggest an ongoing imbalance for the participation of women/females and suboptimal SGR in cardiovascular clinical trials, especially for certain diseases, with a modest positive trend. More women/females in the authorship team correlate with an increased PPR and are associated with an increase in SGR.

**Graphical Abstract:**

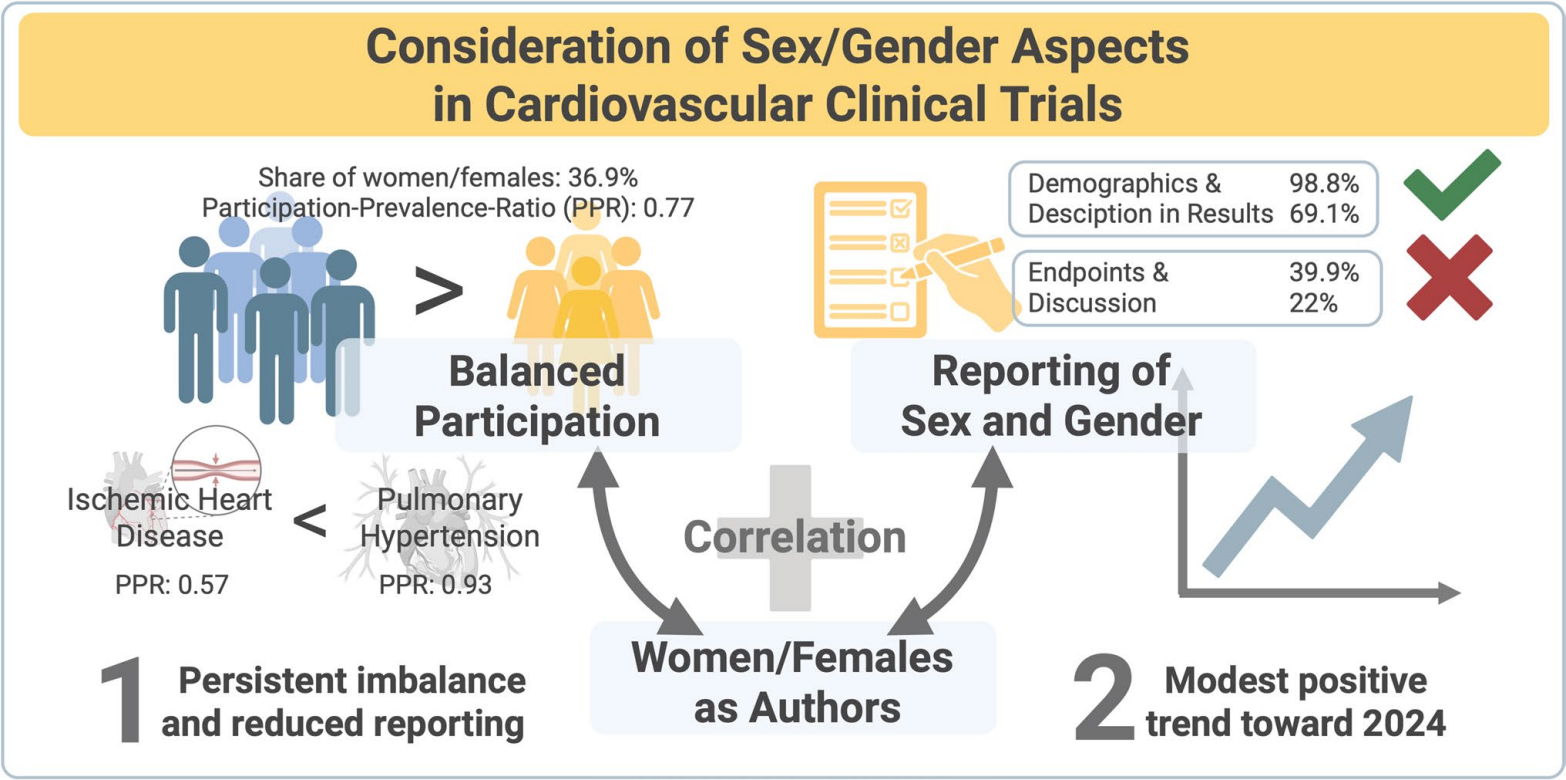

**Supplementary Information:**

The online version contains supplementary material available at 10.1007/s00392-025-02793-3.

## Introduction

Cardiovascular diseases (CVD) are the leading cause of death among both men/males and women/females. Yet, women/females remain underrepresented in cardiovascular clinical trials. A previous assessment of the participation of women/females in completed cardiovascular trials registered in clinicaltrials.gov between 2010 and 2017 showed a median woman/female-to-man/male ratio of 0.51 overall [1]. The ratio remained suboptimal after adjusting for sex/gender-specific prevalence. The weakest representation of women/females was found in trials of heart failure und coronary heart disease. A similar trend was shown in the field of interventions and drug trials with minor signs of improvement [2]. Results of Bastian-Pétrel et al. support these findings by analyzing clinical trials included in the guidelines of the European Society of Cardiology (ESC) for chronic coronary syndromes [3]. Here, they found an association of reduced numbers of women/females as authors, as well as a deficit of sex/gender-sensitive designs. In other fields, studies came to similar conclusions [4]. In recent years, advocacy, and adapted guidelines tried to support a more balanced representation of societal groups, considering not only sex/gender but also race, ethnicity, and age [5]. Despite the increasing initiatives, a recent study describes continuing underrepresentation in cardiovascular clinical trials 2017–2023 [6]. The halting improvements may indicate the need for further sex/gender-sensitive analyses and initiatives in clinical trials to ensure that medical treatments are safe and effective for everyone.

A more balanced participation of women/females and men/males is the basis for conducting representative subgroup analyses. Sex/gender-sensitive reporting (SGR) not only serves to identify relevant differences between subgroups but also allows for mechanistic understanding and hypothesis generation — which could spark future research. Hence, experts developed Sex and Gender Equity in Research (SAGER) guidelines to increase sex/gender-sensitive reporting in research in 2016 [7]. While these guidelines suggest an inclusive use of sex/gender, current evidence suggests a rather infrequent integration after their initiation [8–10]. However, multiple journals promote the need for SGR and urge the use of SAGER guidelines [11, 12]. There is a need for a close, comprehensive and frequent monitoring of different measures to evaluate the status quo and potential improvements. Furthermore, the relationship between a balance in participation and SGR as well as associated factors needs to be evaluated constantly. In the past, especially inclusion of women/females as authors has been shown to correlate with increased participation of women/females in clinical trials [13, 14], while evidence shows a longstanding gap for women/females as senior authors in the life sciences, and specifically cardiovascular research publications [15]. A systematic, large-scale investigation of SGR in clinical cardiovascular trials has not been thoroughly carried out yet.

Therefore, this study aims to longitudinally collect comprehensive information for different fields in cardiovascular clinical research to update previous studies with regards to (1) participation of women/females, and (2) sex/gender-sensitive reporting, as well as (3) the association of women/females as authors in identified trials from 2018 to 2024.

## Methods

### Terminology

The underlying data sources for determining sex/gender of participants and author names are manuscripts. We cannot determine if sex/gender in clinical trials has been determined clinically (through genetic testing or other means) in the investigated studies, or whether the determination was carried out through self-report or name identification, in which case the term sex could be incorrect in a (small) number of case*s.* For authors, name identification was used. In this manuscript, we therefore use the term sex/gender, and refer to our analytical groups as men/males (man/male; m/m), and women/females (woman/female; w/f). We encourage data collection to include more specific categories across sex, gender, and other biological and sociocultural dimensions to better support more exacting analyses crucial to understanding and improving global health.

### Search strategy and study population

**Fig. 1:**
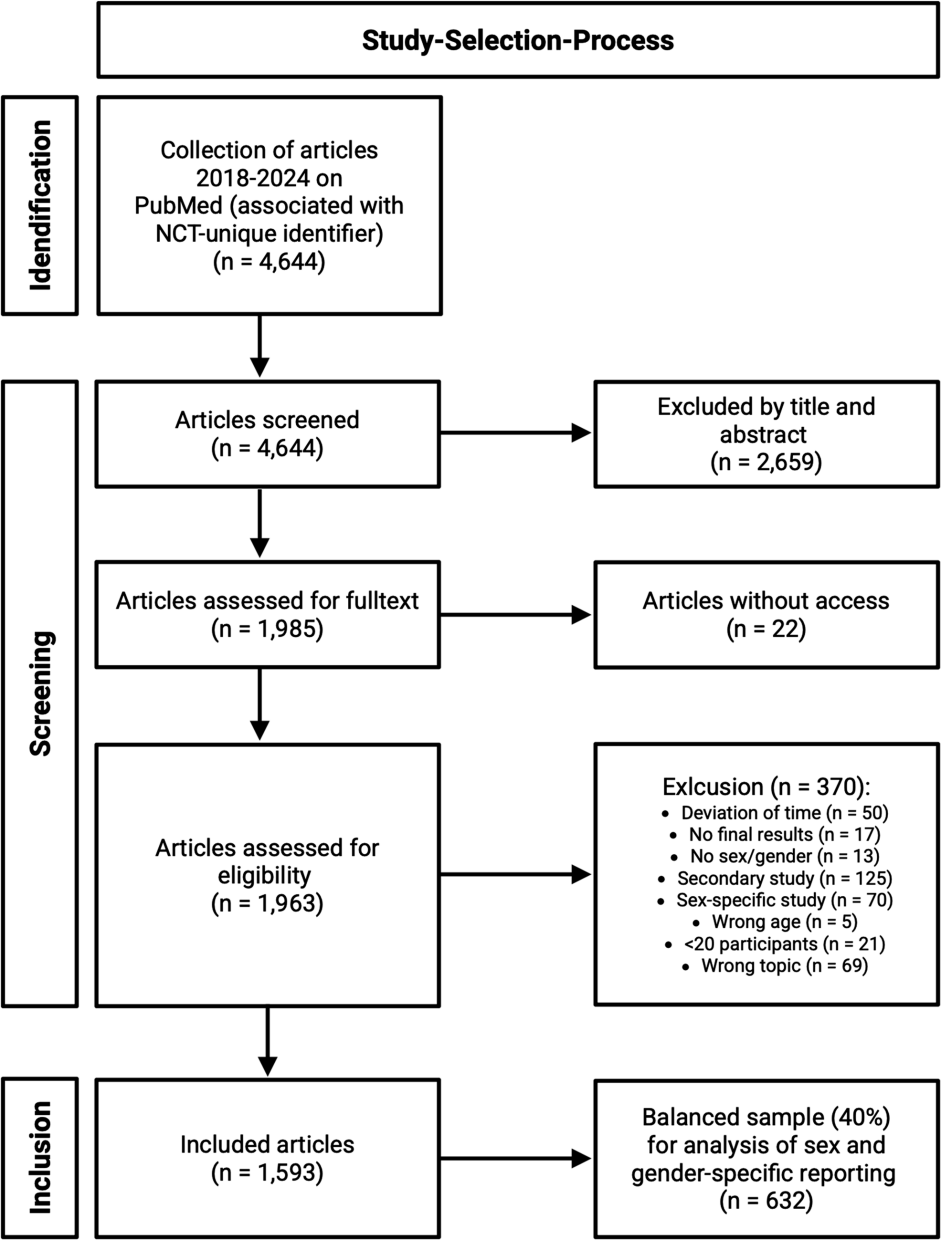
Flow chart of selection process

We used PubMed as data source to identify articles reporting clinical trials and considered articles registered with a national clinical trial (NCT) number for further analyses. In line with previous studies, we included articles following the search terms for Medical Subject Headings (MeSH terms): *cardiovascular diseases*; *heart failure*; *stroke*; *myocardial infarction*; *arrhythmias, cardiac*; *acute coronary syndrome*; *hypertension;* and *hypertension, pulmonary* [1]. To allow full-text analysis, we used PubMed articles as our primary input source as opposed to self-reported results from clinicaltrials.gov. We filtered trials with the electronic publication date between January 2018 and December 2024, which are randomized controlled trials and focused on adults over 18 years. Two reviewers were involved in the screening and eligibility assessment, resolving discrepancies through discussion. We validated extracted data through independent assessment of a random data set (*n* = 100) resulting in high accuracy of 83% before and 96% after resolving differences in discussion. Inclusion and exclusion criteria are summarized in Table S1. Trials were excluded that did not match topics mentioned in the search criteria above or were not directly related to cardiovascular diseases (e.g., hemorrhagic bleeding), or were outside of the publication year range, younger participant’s age (< 19 years), or different publication type. Secondary analyses with repetitive descriptions of the same collective were excluded to reduce a repetition bias. If unclear, the study with the better group description was chosen. Central for evaluation for the publication year were the dates on PubMed. The secondary source ID was collected to ensure registration and reduce duplication. The summary of this process is shown in a flow chart in Fig. 1.

### Data extraction

After passing of the study’s eligibility, the following data was extracted manually: (1) title, (2) NCT-number, (3) disease entity, (4) number of men/males, (5) number of women/females. Further automated extraction from metadata included: (6) publication year and month, (7) names of authors and (8) author affiliation. If necessary, missing data was manually collected. For analysis of SGR, full texts of 40% randomly selected publications were analyzed for the sex/gender-associated words: “gender”, “sex”, “male”, “female”, “men”, “women”, “subgroup” and “adverse” at various locations of the text. The researched disease entity was categorized per trial in “Atrial Fibrillation”, “Cardiomyopathy”, “Diseases of Lower Extremity”, “Endocarditis and Valve Diseases”, “Other CVD”, “Heart Failure” (HF), “Ischemic Heart Disease” (IHD), “Pulmonary Hypertension” (PH), “Stroke” and “Systemic Hypertension”. The category “Other CVD” was used when there was no focus on one specific CVD, multiple foci of different CVDs or a general population of participants with unknown prevalence of CVD.

### Endpoints

The primary endpoint of this study is the representation of women/females in identified trials measured by share of participation and participation-prevalence-ratio (PPR). The secondary endpoint is the level of sex/gender-sensitive reporting (SGR) in clinical trial publications with a special focus on results and discussion sections. The third endpoint is the association of women/females as authors with the participation of women or SGR.

### Analysis of participation and calculation of PPR

Participation was summarized by the absolute number of participants, relative share of women/females (percentage) and PPR. To calculate the PPR, the share of women/females as participants per trial was divided by a ratio of prevalences for women/females and men/males specific for disease entities, location, and year as previously reported [1, 3, 4]. A PPR ranging from 0.8 to 1.2 was considered an optimal representation of women/females.

### Determination of regions from author affiliation for region and disease specific prevalence

To calculate the PPR, location-specific prevalences of disease entities for women/females and men/males were required. We linked the location of articles by analyzing the reported author affiliations and assumed the location with the majority of the reported affiliations. A large language model (LLM, Chat GPT-4o) was used to evaluate and integrate affiliation data collected from PubMed through an annual baseline XML dataset. Regions were summarized in the World-Bank-Organization (Europe, Northern America, Latin America and Caribbean, Northern Africa and Western Asia, Sub-Saharan Africa, Central and Southern Asia, Eastern and South-Eastern Asia, Oceania) aligned with the region-specific statistics from the global burden of disease (GBD) database [16]. Manual validation of 200 affiliation strings showed an accuracy of 100% for the LLM designation.

The determined location was used to extract region- and year-specific prevalence estimates from the GBD database [16] and relevant publications. When data for a disease entity were not available through GBD, estimates were obtained from publications. Literature was also used for heart failure and its classification in preserved (HFpEF) and restricted ejection fraction (HFrEF) as those are not listed as causes in the GBD. The prevalence estimates for women/females with HF without specification (0.5), HFrEF (0.42) and HFpEF (0.57) were assumed [17].

### Analysis of SGR

The analysis of SGR focused on the descriptive part of an article following the original SAGER criteria (including title, abstract, results, discussion). SGR was analyzed in a random subsample (40%) of all articles which was created to have a better comparison with a balanced number of articles and proportion of research foci throughout the years. The overall composition of disease entities varied less than 2% compared to all trials and was consistent within the years of the observation period (Fig. S1). Following, full text subsections were searched for words associated with sex/gender. SGR in title and abstract was analyzed relatively compared to the entire subsample, SGR of adverse events relatively to trials including analysis of adverse events in general. SGR of results and discussion was analyzed summarizing four different subsections: (1) demographics (e.g., descriptive table with stratified gender or sex proportions in methods or results), (2) in-text mention in methods or results, (3) within their observation of an endpoint including subgroup analysis and adjustment for confounding, and (4) discussion including limitations. Supplementary material was checked when available and mentioned.

### Determination of author sex/gender

For analyses of the influence of author sex/gender, sex/gender designation by name was carried out using a local opensource LLM (Gemma2 27B with Ollama). As a limitation, only authors with a first name could be recognized. Compared to a manual validation of 668 author names, the used approach reached high accuracy (92.8%) with minor disadvantages for names of Asian heritage. Taken together, the various validation exercises indicated the LLMs to be valid approaches for scaling location and sex/gender designation for our encompassing dataset similar as indicated before [18].

### Data processing and statistical testing

After calculation of PPR, standard descriptive statistics summarized share of women/females and PPR for each trial individually and together represented by median and 95%-confidence intervals (95%-CI). For the longitudinal analysis of trials, the shares of women/females from every trial were plotted against time (month and year). Linear regression was used as well as Pearson correlation to summarize the longitudinal trends, reporting the Pearson correlation coefficient “r” and *p*-values for statistical significance.

For SGR, mention of all articles was summarized within the four subsections. The collective mention of SGR within one article was also reflected by the sum of these four subsections (0–4) and shown relatively compared to all articles within a year or disease entities. The sum of articles with at least three positive subsections was used as an estimation of ensured SGR in endpoint or discussion.

The sex/gender of authors was analyzed for different authorship positions (first and last) as well as relative proportion of women/females compared to all authors. A correlation of PPR and proportion of women/females as authors was performed. Shares of SGR at four subsections was shown stratified for women/females as first and/or last author. Furthermore, the collective mention of SGR within one trial was shown relatively compared to all trials stratified by women/females as first and/or last author, women/females as first author, last author, both authors, and only men/males, as well as three different subcategories of PPR (< 0.8; 0.8–1.2; > 1.2). Statistical analysis for PPRs with women/females as first and last authors was done by Mann–Whitney test. Binary categories were tested for significance using Chi-Square test. A multiple logistic regression model was utilized to measure potential associations of author gender, PPR and SGR. Women/females as first and/or last authors (vs. men/males as first and last authors) and a PPR within the optimal range of 0.8 and 1.2 (vs. outside this range) were used as binary independent variables and the sum of SGR ≥ 3 (vs. SGR < 3) as binary outcome. To estimate the crude association of each independent variable with the outcome variable, two univariate regression analyses were performed separately. The results were reported as odds ratios (OR) with 95%-CI and *p*-values. For all experiments, a *p*-value < 0.05 was considered as statistically significant.

For graphical and statistical testing, the statistical environment R (version 4.1.2)/R-Studio (Version 2024.12.1 + 563), Python (version 3.9.20 with country_converter library to standardize country names to ISO3 codes) and PRISM (version 10.4.1) were used. The graphical abstract and Figure 1 were edited with biorender.com.

## Results

### Characterization of study populations

A total of 1593 articles that published results of clinical trials were systematically selected after an initial screening of 4644 articles (Fig. 1). Overall, most of the trials focused on IHD (21.2%) or had an undefined/general focus (23.2%), followed by stroke (18.1%), HF (11.7%), and systemic hypertension (11.2%) (Table 1). Other focal areas represented less than 10% of included articles. The highest number of participants overall was found in trials focused on IHD, while the lowest number of trials focused on PH (Table 1). Trials were mostly located in North America (36.7%) and Europe (37.1%), followed by trials from Eastern and South-eastern Asia (17.8%). The proportion of trials from Latin America, Africa and Oceania was collectively under 10% (Table 1).

**Table 1:** Description and number of participants of included studies

Group	Number of studies (%)	Sum of women/female participants/ Total participants (%)
Total	1593 (100)	716,569/1,859,736 (38.5)
Year of publication	1593 (100)	716,569/1,859,736 (38.5)
2018	290 (18.2)	152,184/427,274 (35.6)
2019	265 (16.6)	125,146/318,213 (39.3)
2020	279 (17.5)	64,067/187,893 (34.1)
2021	218 (13.7)	109,546/272,434 (40.2)
2022	124 (7.8)	118,822/263,316 (45.1)
2023	136 (8.5)	44,741/119,508 (37.4)
2024	281 (17.6)	102,063/271,098 (37.6)
Researched disease entities	1593 (100)	716,569/1,859,736 (38.5)
Ischemic heart disease	337 (21.2)	156,862/559,595 (28.0)
Different focus on CVD	369 (23.2)	245,371/551,316 (44.3)
Stroke	289 (18.1)	72,629/189,563 (38.3)
Systemic hypertension	179 (11.2)	55,812/123,178 (45.3)
Heart failure	186 (11.7)	58,257/155,470 (37.5)
Atrial fibrillation	119 (7.5)	104,002/225,393 (46.1)
Diseases of lower extremities	42 (2.6)	5546/16,969 (32.7)
Valve diseases and endocarditis	47 (3.0)	16,114/34,950 (46.1)
Pulmonary hypertension	25 (1.6)	1976/3302 (59.8)
World Bank regions	1593 (100)	716,569/1,859,736 (38.5)
Europe	591 (37.1)	176,479/487,242 (36.2)
North America	585 (36.7)	371,953/923,290 (40.3)
Latin America and Caribbean	57 (3.6)	9620/24,464 (39.3)
Northern Africa and Western Asia	35 (2.2)	3199/8660 (36.9)
Sub-Saharan Africa	4 (0.3)	617/1151 (53.6)
Central and Southern Asia	15 (0.9)	9289/31,448 (29.5)
Eastern and South-Eastern Asia	283 (17.8)	115,715/324,749 (35.6)
Oceania	23 (1.4)	29,697/58,732 (50.6)
Trial size (quartiles)	1593 (100)	716,569/1,859,736 (38.5)
First (≤ 72)	399 (25.0)	6819/16,955 (40.2)
Second (72 < × ≤ 182)	400 (25.1)	18,665/47,780 (39.1)
Third (183 < × ≤ 617)	396 (24.9)	49,896/128,890 (38.7)
Fourth (> 617)	398 (25.0)	641,189/1,666,111 (38.5)
Women/females as first and/or last author	1593 (100)	716,569/1,859,736 (38.5)
Yes	789 (49.5)	336,715/815,604 (41.3)
No	804 (50.5)	379,854/1,044,132 (36.4)
Sex/gender-sensitive reporting	632 (39.7)	310,349/769,761 (36.3)
Less than 3 of 4 subsections	384 (60.8)	79,596/237,907 (33.4)
At least 3 of 4 subsections	248 (39.2)	230,753/531,854 (43.4)

*CVD* (cardiovascular diseases)

### Participation of women/females

**Fig. 2:**
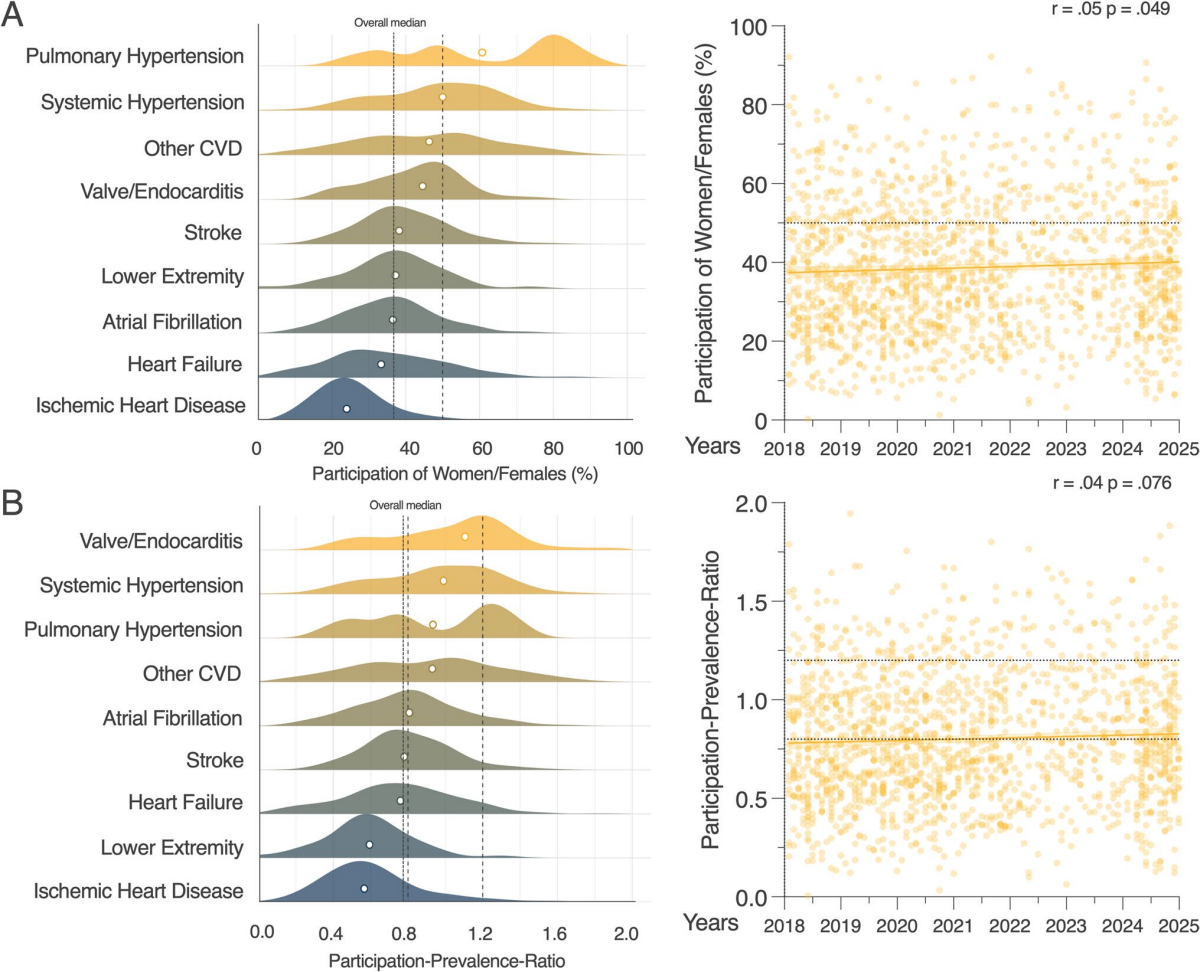
Participation of women/females in clinical cardiovascular trials by year and disease entity. Summary of **A** share and **B** participation-prevalence-ratio (PPR) for disease entity (left) and years of publications (right) of 1593 identified trials. Indicated are parity (50%) and PPR (0.8–1.2), overall median as well as correlation by Pearson with calculated correlation coefficient (*r*) and assumed significance (*p* < 0.05)

Women/females accounted for 38.5% of 1,859,736 total participants across all investigated trials (Table 1). When analyzing the participation of women/females per trial (Fig. 2A), we find a median share across all trials to be 36.9% (95%-CI: 35.8, 37.8). We next investigated the median share for each disease category and found a representation of over 50% for women/females in clinical trials for PH and systemic hypertension, while the distribution was relatively heterogenous. The median share for trials investigating other disease entities was under 50% with the minimum in IHD where women/females accounted for 24.0% (95%-CI: 23.4, 25.4) of the participants. From 2018 to 2024, we found a small, but statistically significant, increase of women/females as participants in cardiovascular clinical trials overall (*r* = 0.05, *p*-value = 0.049). The highest median share of women/females was found in trials with the majority of authors from Sub-Saharan Africa with over 50%, while all other regions remained under 50% (Supplementary Fig. 2A).

To better account for the expected or optimal number of women/females as participants in trials, we adjusted for the location and disease specific prevalence of women/females (Fig. 2B) by calculating the PPR (desired ratio between 0.8 and 1.2). We found that the median PPR overall was 0.77 (95%-CI: 0.74, 0.79), which means that PPR remained below the optimal range. The lowest median PPR was found in trials focusing on IHD with 0.57 (95%-CI: 0.54, 0.60) followed by diseases of the lower extremity with 0.59 (95%-CI: 0.52, 0.66). The median PPRs of heart failure, stroke, or atrial fibrillation trials were at the lower limit of optimal balance while trials in the categories Other CVD, PH, systemic hypertension and valve or endocarditis were within the optimal range. PPR remained stable between the years 2018 and 2024 (*r* = 0.04, *p*-value = 0.076). The median PPRs for trials from Eastern and South-Eastern Asia, Central and Southern Asia as well as Europe were suboptimal, while all other regions were within the optimal range (Supplementary Fig. 2B).

### Sex/gender-sensitive reporting

**Fig. 3:**
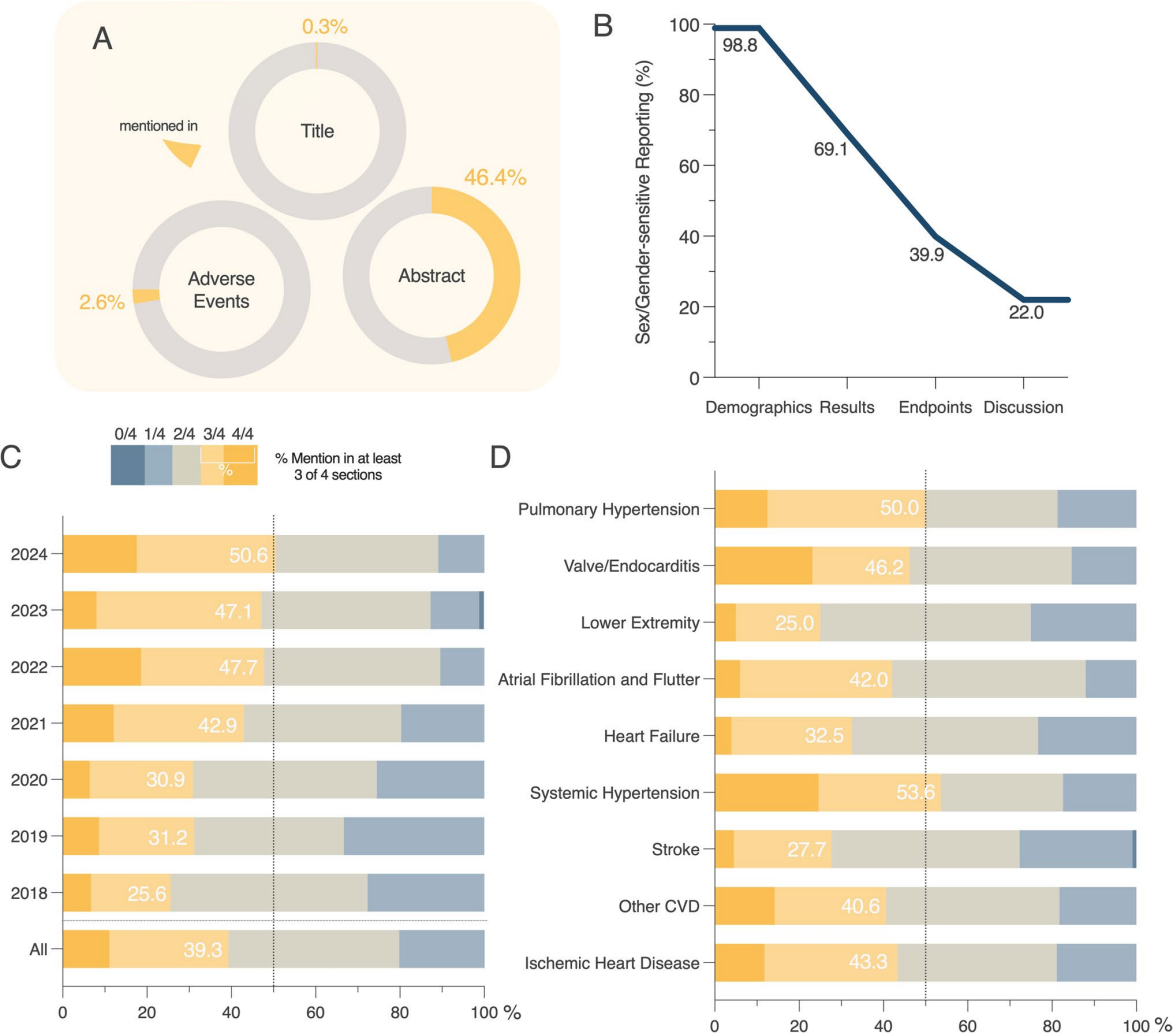
Sex/gender-sensitive reporting (SGR) in a balanced sample of clinical cardiovascular trials by year and entity. SGR was determined by analyzing the use of sex/gender-sensitive words (“sex”, “gender”, “male”, “female”, “women”, “men”) in publications and are shown proportional for indicated sections **A**, **B**. *Demographics* include any indication of sex/gender of the participants within a study demographics table, while *results* imply a mention within the results section of the text. *Endpoints* include subgroup analyses, stratification, and adjustment of regression by sex/gender, while *discussion* include mentions in the discussion section, including limitations. Categories that reported sex/gender were summarized and grouped by publication year **C** and disease entities **D**. Numbers indicate the share of trials with SGR in at least three of four sections. Abbreviations: CVD (cardiovascular diseases)

For the analysis of SGR, a subsample of 632 studies was randomly drawn and further balanced for disease entity over the publication period and within each year to reduce a distribution bias and increase comparability (Supplementary Fig. 1A–B). Title, abstract, results, discussion and adverse events were evaluated. Overall, 0.3% of analyzed articles mentioned sex/gender-sensitive words (“gender”, “sex”, “male”, “female”, “men”, “women”) in the title (Fig. 3A). Almost half (46.4%) of the articles specified sex/gender in the abstract. 2.6% of articles that mentioned adverse events stratified these explicitly for sex/gender (Fig. 3A). When analyzing the main manuscript and supplemental materials, we found 98.8% of the articles mentioned the sex/gender of the included trial population in a demographics table (Fig. 3B). 69.1% of articles mentioned sex/gender in the results section and 39.9% considered sex/gender in stratified endpoint analyses, including subgroup analyses (Fig. 3B). 22.0% of articles used sex/gender-sensitive words in the discussion (inclusive of mention as limitations) (Fig. 3B). Of all analyzed articles, 11.1% used sex/gender-sensitive words in all four sections, and 39.3% of articles reported in at least three of four sections (Fig. 3C). However, we found an increase of the number of articles that reported sex/gender-sensitive words in at least three sections over time, from 25.6% in 2018 to 50.6% in 2024 (Fig. 3C). Separating articles by researched disease entity reveals that only 27.7% of articles about stroke, 25.0% of articles about peripheral artery diseases, and 32% of articles about heart failure report sex/gender-sensitive words in at least three of four sections (Fig. 3D). In 53.6% of articles that focus on systemic hypertension and 50.0% of articles that focus on pulmonary hypertension sex/gender-sensitive words were reported in at least three of four sections (Fig. 3D). Articles about valve diseases and endocarditis, IHD, other CVD, and atrial fibrillation/flutter mentioned sex/gender-sensitive words in at least three of four sections between 40.6% and 46.2% (Fig. 3D). Regional analyses showed no relevant differences in SGR (Supplementary Fig. 2C).

### Associations of women/females as authors and participation

**Fig. 4:**
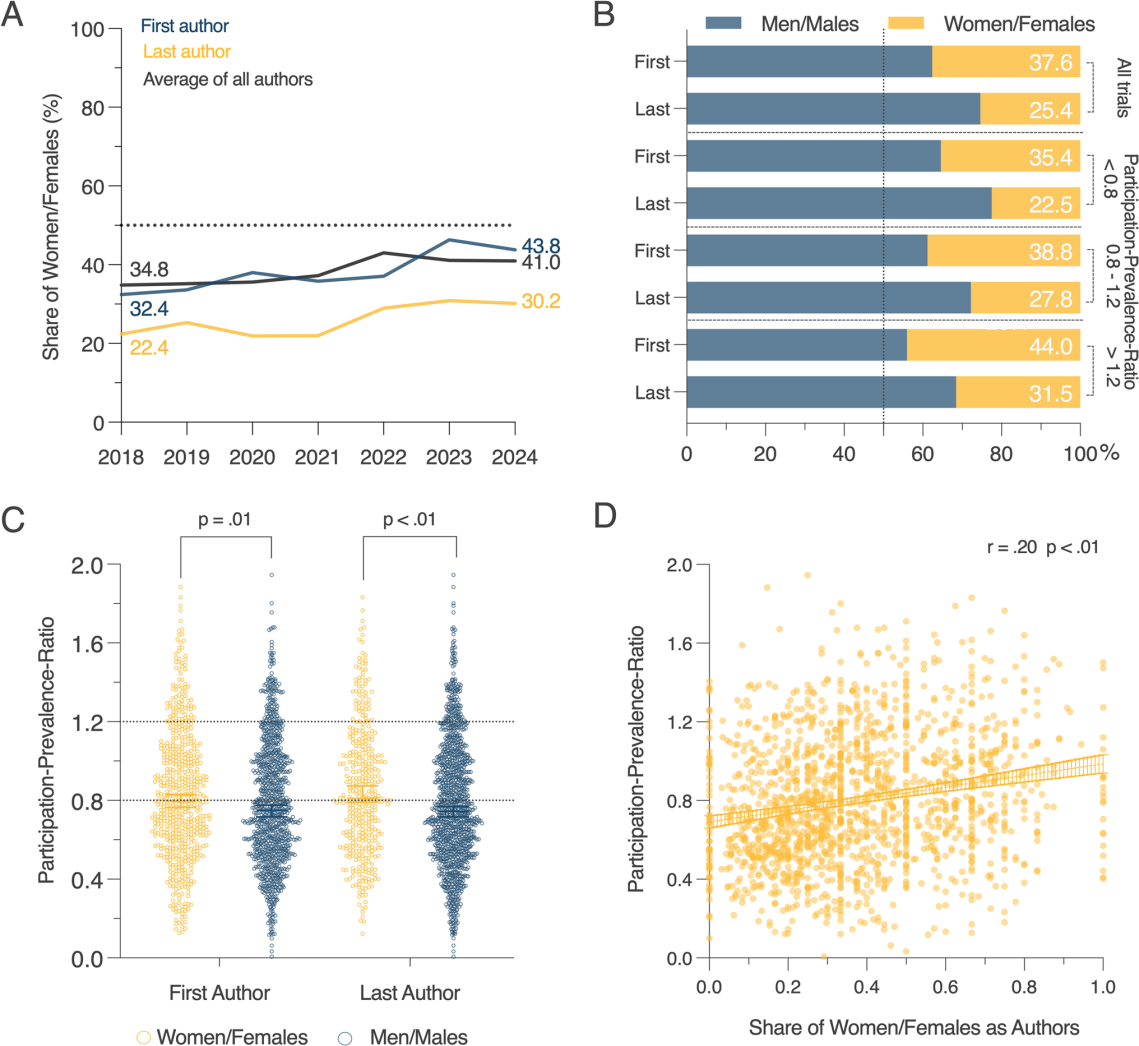
Association of women/females as authors with a balanced participation. Summarized are the association of women/females as authors in different authorship positions (first, last and average of all authors) with the number of women/females as trial participants. Indicated are the proportion of respective authorships over time **A** and the participation-prevalence-ratio (PPR); suboptimal < 0.8 or > 1.2, and optimal (0.8–1.2) **B**. Association of women/females as first and last authors with PPR **C** and association of the share of women/females among authors with PPR **D**. Mann–Whitney test **C** and Pearson-correlation with correlation coefficient (*r*) **D** were used with an assumed significance when *p* < 0.05

It was previously suggested, that the number of women/females as participants of clinical trials (e.g., HF trials) correlates with the number auf women/females on the research team, represented as authorships on articles, especially in leading roles (first and last authorships) [13, 14]. Therefore, we determined the sex/gender of first, last, and interior authors of included articles and found that overall, the share of women/females in first (32.4% vs. 43.8%) and last (22.4% vs. 30.2%) authorship positions, as well as the total share of women/females as authors on trial publications (34.8% vs. 41.0%) increased between 2018 and 2024 (Fig. 4A).

On average, 25.4% of articles were authored by a woman as last author, while 37.6% of articles were authored by a woman as first author (Fig. 4B). Next, we investigated trials in which the PPR was above 1.2 and found that more women/females were first and last authors on these articles (44.0% and 31.5%) respectively, while when the PPR was in the range of 0.8–1.2, 27.8% of articles had women/females as last authors and 38.8% of articles had women/females as first authors (Fig. 4B). Articles with a PPR < 0.8 had the lowest share of women/females as first and last authors (35.4% and 22.5%). The median PPR of trials with women/females as first or last authors was significantly higher compared to trials with men/males as first or last authors (Fig. 4C). We found the share of women/females as authors and the PPR modest, but positively and statistically significant correlated (*r* = 0.2, *p*-value < 0.01) (Fig. 4D).

### Association of women/females as authors and sex/gender-sensitive reporting

**Fig. 5:**
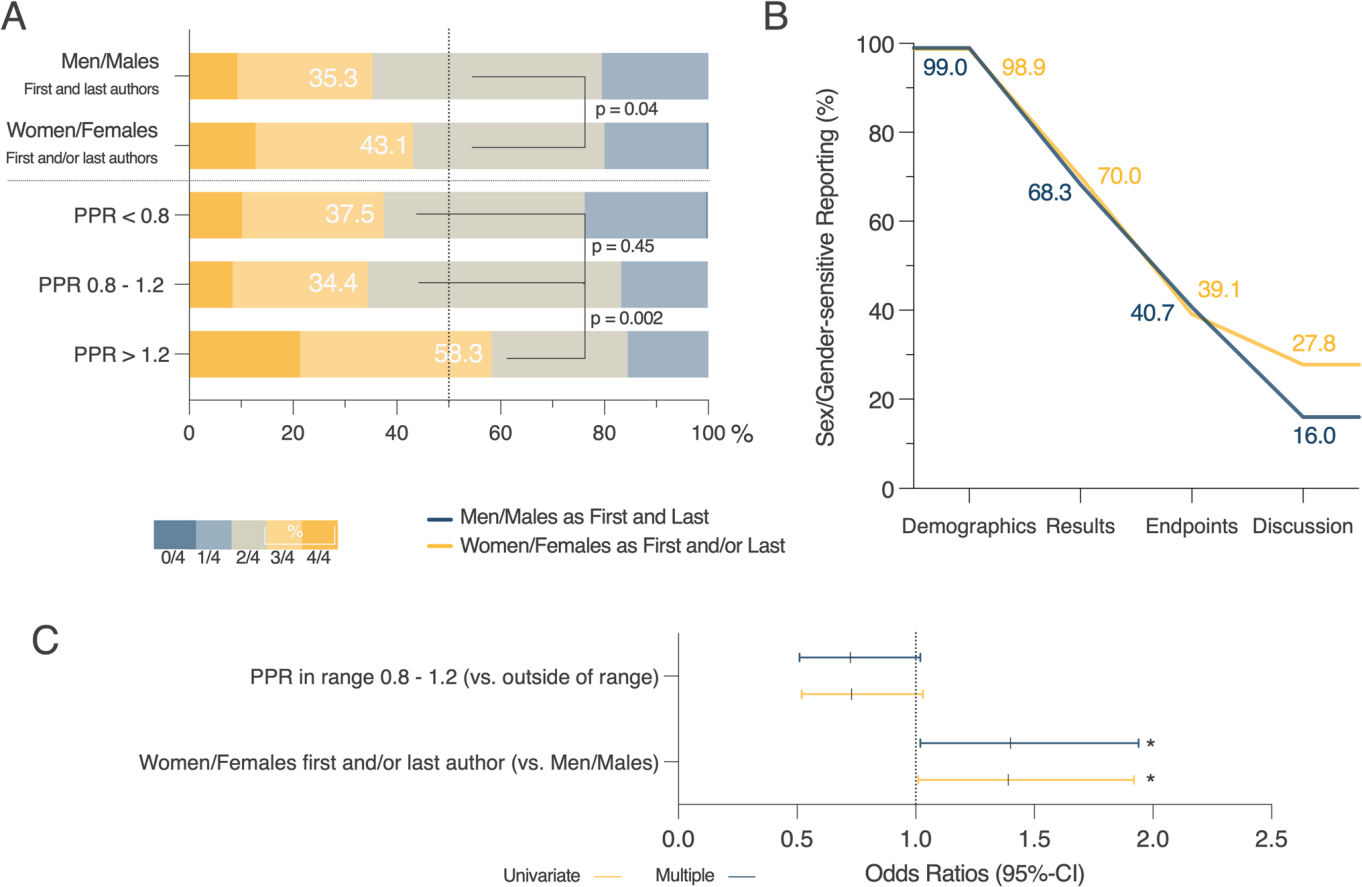
Association of women/females as authors with sex/gender-sensitive reporting (SGR). The sections reporting sex/gender within each trial were summarized and shown proportionally for articles with women/females as first and/or last authors, men/males as first and last authors, and participation-prevalence-ratio (PPR). Numbers in bars indicate the proportion of trials with SGR in at least three of four parts and were compared by statistical analysis with Chi-square test between indicated groups **A**. Comparison of SGR as percentage in specified sections of the publications analyzed across articles with women/females as first and/or last author compared to men/males as first and last author **B**. Univariate and multiple logistic regression model evaluating the association between an optimal PPR (0.8–1.2 vs. suboptimal < 0.8 and > 1.2), authorship (women/females as first and/or last author vs. men/males as first and last author) and SGR (≥ 3/4 vs. < 3/4 sections in articles). Shown are odds ratios and 95%-confidence intervals (95%-CI). Statistical significance was assumed when *p* < 0.05 and shown by an asterisk (*) **C**. Abbreviations: PPR (Participation-Prevalence-Ratio)

We investigated whether women/females as authors have an influence on SGR in clinical trials. We first found that articles with women/females in any leading author position (defined as first or last, or both first and last author positions) showed a higher likelihood of SGR in at least three out of four article sections when compared to men/male-led studies (i.e., men/male first and last authorship) (43.1% vs. 35.3%, *p*-value = 0.04) (Fig. 5A). We further asked whether SGR was influenced according to a study’s PPR being in optimal range (0.8–1.2) or out of range (< 0.8 and PPR > 1.2) and found that the proportion of articles with SGR in at least three of four sections was increased significantly when PPR was above the optimum range when compared to optimal PPR (58.3% vs. 34.4%, *p*-value = 0.002) (Fig. 5A). There was no significant difference between articles with suboptimal and optimal PPR (*p* = 0.45). Because women as authors were represented to a higher degree in articles with above-optimal PPR (Fig. 4B), and SGR was increased when women were in a leading author position (Fig. 5A), we further investigated which sections of an article were relatively more considered. This is particularly interesting given that external requirements (e.g., journal regulations) enforce SGR, however, the quality of the reporting depends on endpoint reporting and discussion. We found higher SGR in the discussion section of manuscripts when women/females were in a leading author position (first and/or last author) of the trial publication when compared to men/males-led studies (27.8% vs. 16.0%) (Fig. 5B). We found no relevant difference in SGR in demographics, results, and endpoints when comparing the two groups (Fig. 5B).

A multiple regression model was used to strengthen this analysis between women/females as first and/or last authors (independent variable vs. men/males as first and last author as reference), a PPR within the range of 0.8–1.2 (independent variable vs. not in range) and SGR of three or more sections (dependent variable vs. SGR in two or fewer sections as reference) (Table S2). In this analysis, the association of women/females as first and/or last author with SGR of three or more sections was shown with an OR of 1.4 (95%-CI: 1.02, 1.94, *p*-value = 0.04) (Fig. 5C). The PPR in an optimal range was not significantly associated with a higher SGR (Fig. 5C).

## Discussion

This study supports evidence of the persistent underrepresentation of women/females in clinical cardiovascular trials between 2018 and 2024, although an overall upward trend in participation of women/females could be observed over time. Crucially, this study identifies a continued deficiency in sex/gender-sensitive reporting, particularly in the reporting and discussion of endpoint data. In addition, women/females also remained underrepresented as authors of the investigated studies, particularly in leadership roles. To our knowledge, this is the first large, longitudinal study to observe and examine the relationship between participant representation, SGR, and women/females as authors across diverse cardiovascular subspecialties. Importantly, we found that women/females in lead authorship positions were significantly associated with an increased SGR and PPR.

Our findings align with previous reports documenting the underrepresentation of women/females in cardiovascular trials, including those that underpin major clinical guidelines for chronic coronary artery disease and hypertension [3, 4]. Prior analyses have similarly highlighted participation gaps, yet lacked consideration of SGR [1, 6]. Unlike studies limited to clinicaltrials.gov data, we focused on published trials linked to a ClinicalTrials.gov identifier (NCT-ID), minimizing selection bias associated with incomplete or self-reported registries [1, 6, 19]. The persisting underrepresentation of women/females in clinical trials has been the subject of extensive discussion and reasons are manifold [6, 19–21]. For example, on the individual level, the hesitation to participate might stem from a lack of trust, limited knowledge about clinical trials, fear of side effects, logistical barriers (most importantly time), and reduced disease awareness. On the institutional level, fewer referrals, financial constraints, lack of awareness, and missing information about the importance of sex/gender in clinical trials might play a role, as well as the underrepresentation of women/females in trial leadership [20, 21]. Proposed solutions include raising standards in communication, health education and advertisement, as well as specifying study protocols that allow for delays to ensure adequate enrollment of women/females or the removal of unnecessary exclusion criteria [20, 21]. In addition, regional differences of PPR in our analyses, particularly the low participation in trials led by a majority of authors in Asia, may indicate socioeconomic and cultural factors. Our study highlights that the research community might need to improve trial planning, execution and peer-review to better serve the population. Nevertheless, such standards must be applied universally, as the modest positive trends indicate improvements remain slow to emerge.

Furthermore, consistent with previous smaller-scale analyses, our qualitative review revealed a substantial decline in SGR throughout the research process from data collection to analysis and discussion. Only 39.3% of trials demonstrated sex/gender-specific disaggregation of endpoints in the published report or discussion inclusive of the mention within their limitations, with notably low reporting rates in peripheral artery disease, stroke, and heart failure trials. In contrast, the highest levels of SGR were observed in studies on systemic and pulmonary hypertension. Notably, adverse events were disaggregated by sex/gender in just 2.6% of all studies reporting these events. These patterns underscore that while women/females are included in most trials, sex/gender-specific data are rarely fully analyzed or discussed.

This study has several limitations inherent to its descriptive and observational design. While we assessed associations, we cannot draw causal conclusions. The sample was limited to published trials with both a ClinicalTrials.gov registration and a linked PubMed publication, potentially introducing selection bias by excluding unpublished or unsuccessful studies. Field-level comparisons may be affected by structural differences between specialties, including variations in publication practices, geographic distribution, and clinical focus. Furthermore, our reliance on large-language models for determining author sex/gender and affiliation, while validated against human raters, may introduce classification errors. Only primary trial publications were included, likely underestimating the extent of SGR reported in secondary (subgroup) analyses. Finally, although the trends observed are encouraging, the overall low baseline levels of participation and SGR mean that even modest improvements require cautious interpretation.

Despite recent improvements, underrepresentation of women/females as trial participants and lead authors remains a systemic issue in cardiovascular research. Furthermore, SGR remains suboptimal across all subspecialties, limiting the generalizability and equity of clinical trial findings. Our findings suggest that increased involvement of women/females in trial leadership may improve SGR, while an optimal participation may not. This implies that policies to enhance equity might need to address these elements in tandem. Current guidelines on sex/gender inclusion may not yet be fully adopted due to the lengthy planning timelines and rigid protocols of clinical trials. However, the observed upward trends in participation and authorship of women/females, and SGR — though modest — are promising. The demonstrated importance of women/females as authors for a balanced participation and SGR underscores the critical need to further strengthen equity in medicine and research. Local initiatives and support programs have been central to this progress, including mentorship and network opportunities, flexible funding schemes that facilitate the balance of family and career, and the implementation of regulations and quotas in application processes. These measures have likely contributed to the positive trend observed over the years [22, 23]. At the same time, measures are needed to address the ongoing pay/funding/promotion gaps, improve career transparency, flexible working models (including shared leadership and part time work models), and fair maternity and parental leave options. Furthermore, leadership training for current and future leaders might help to provide a comprehensive, evidence-based overview, including sex/gender-based biases and discrimination. This responsibility is particularly urgent at a time when equity, diversity and inclusion efforts may be under threat. The present study highlights the need for coordinated action across research institutions, journals, funding bodies, and policy frameworks, as well as the importance of close monitoring to ensure continued progress.

## Supplementary information

Below is the link to the electronic supplementary material.

ESM 1 (PDF 2.15 MB)

## Data Availability

The data that support the findings of this study are available from the corresponding author upon reasonable request.
