# Supplementary material for: ​Consideration of sex/gender aspects in cardiovascular clinical trials
Source: Clin Res Cardiol. 2025 Nov 26;114(12):1752–64. doi: 10.1007/s00392-025-02793-3 (PMC12708832; doi:10.1007/s00392-025-02793-3)
Supplement: Supplementary file 1 — (PDF 2.15 MB) [file 392_2025_2793_MOESM1_ESM.pdf]

Wagistr. 12
8952 Schlieren

Supplement: Consideration of Sex/Gender Aspects in Cardiovascular Clinical Trials

Bley, Maximilian, Mathez, Linda, Menz, Susanne, Stephan, Isabella, Lerchenmueller, Marc, Regitz-Zagrosek, Vera, Lerchenmüller, Carolin

PROTOCOL – Consideration of Sex/Gender Aspects in Cardiovascular Clinical Trials

Target answerable research question: Is there a significant difference in women participation or gender-sensitive reporting in cardiovascular clinical trials from 2018 and 2024?

PICOTS:

- P (Participants): Adult patients participating cardiovascular clinical trials;
- I (Intervention): Balanced participation between sexes and sex/gender-sensitive reporting;
- C (Comparison): Between male and female patients;
- O (Outcomes): Participation-Prevalence-Ratio of different fields (primary endpoint), SAGER-criteria, sex of authors
- T (Time-frame): between 2018 and 2024;
- S (Setting): PubMed (Medline) with connection to Clinicaltirals.gov.

Methods:

Data foundation:

- Cardiovascular clinical trials listed in PubMed registered on clinicaltrials.gov
- Primary screening of Title and Abstract

Search of Pubmed

(("cardiovascular diseases"[MeSH Terms]
OR "heart failure"[MeSH Terms]
OR "stroke"[MeSH Terms]
OR "myocardial infarction"[MeSH Terms]
OR "arrhythmias, cardiac"[MeSH Terms]
OR "coronary disease"[MeSH Terms]

OR "acute coronary syndrome"[MeSH Terms]
OR "hypertension"[MeSH Terms]
OR "hypertension, pulmonary"[MeSH Terms])
AND 2018/01/01:2024/12/31[Date - Publication]
AND "clinicaltrials.gov"[Secondary Source ID]
AND "randomized controlled trial"[Publication Type])
AND (alladult[Filter])

Table S1: Inclusion and Exclusion Criteria

	Inclusion Criteria	Exclusion Criteria
Time	01/01/2018 - 12/31/2024	Before 01/01/2018 and after 12/31/2024
Language	English	No available translation
Text	Full text	No full text
	Association to clinicaltrial.gov	Multiple Publication to the same trial cohort
		No published sex/gender characteristics
Population	Adults over 18 years	Adults under 18 years
	Cardiovascular Diseases	Other focus then CVD
	> 20 participants	< 20 participants
Design	RCT	Other then RCT

Abbreviations: RCT (randomized controlled trial), CVD (cardiovascular disease)

Table S2: Data from multiple regression model for sex/gender sensitive reporting

Predictor	Coefficient (β)	Odds Ratios (95%-CI)	p-value
Intercept	-0.51	0.60 (0.46, 0.78))	< 0.001
Women/females as first and/or last author (vs. men/males)	0.34	1.40 (1.02,1.94)	0.04
Optimal participation-prevalence ratio 0.8-1.2 (vs. non-optimal)	-0.32	0.73 (0.51,1.02)	0.07

Abbreviations: CI (confidence interval)

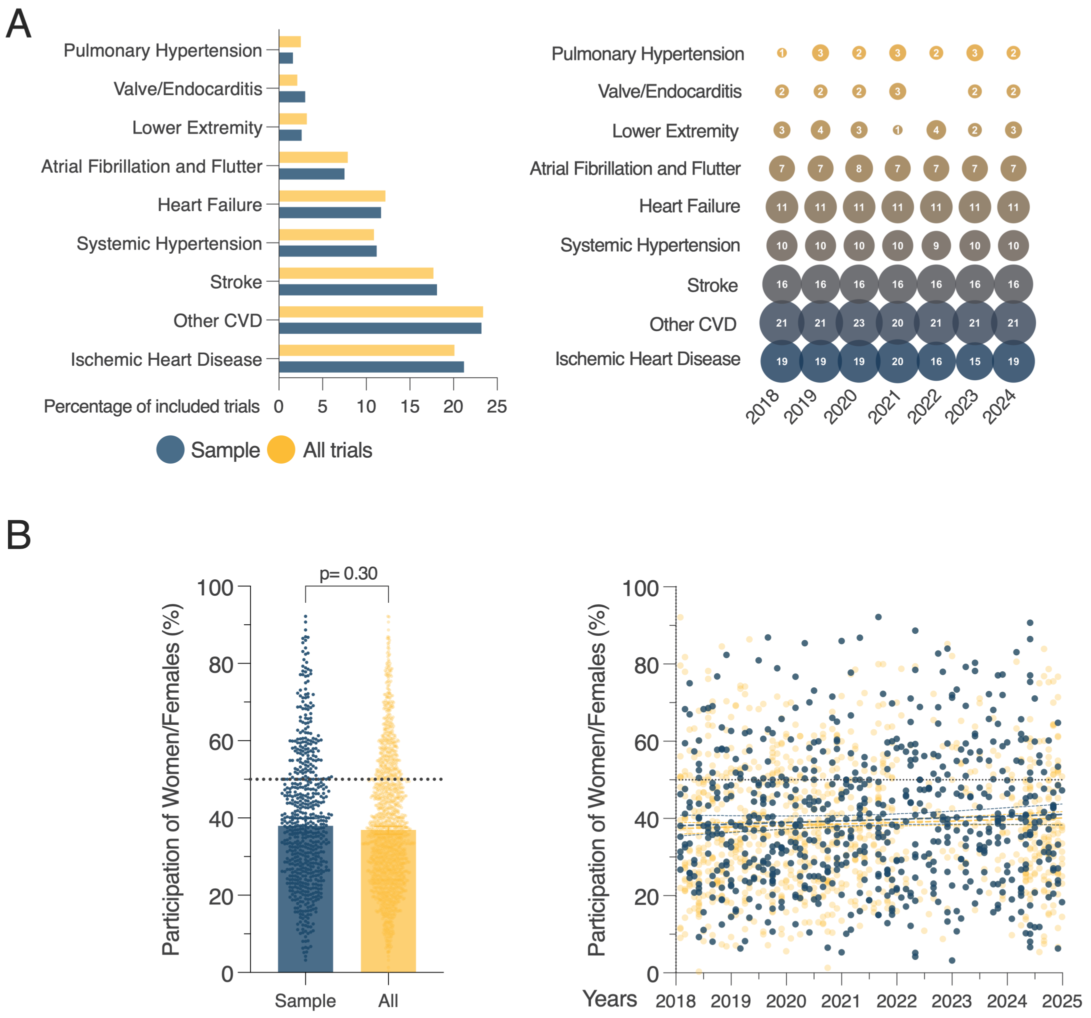

Figure S1: Characterization of balanced subsample. Comparability of sample (n= 632) and total included trials shown by similar distribution of disease entities during different years of publication (A) and share of women/females participating in total and throughout the years of publication (B). Mann-Whitney test comparing indicated groups with an assumed significance $p<0.05$.

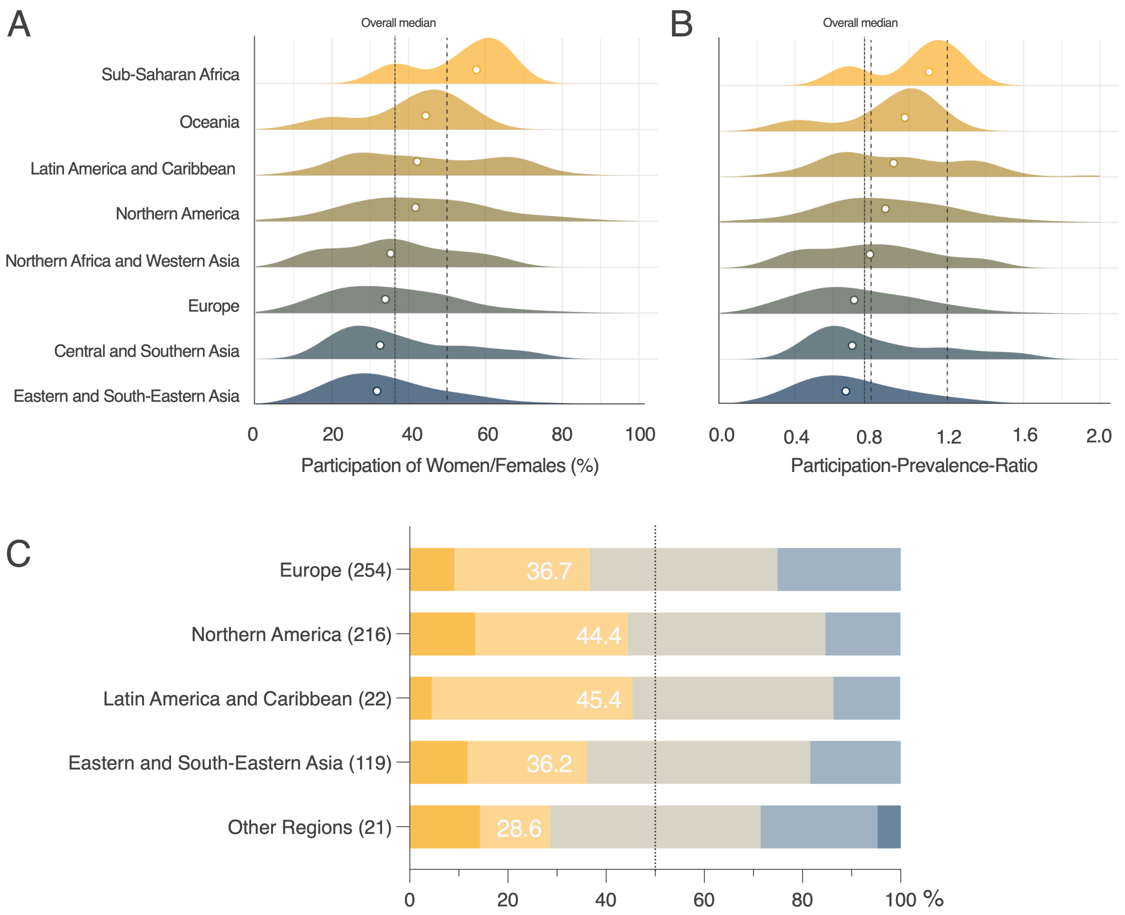

Figure S2: Participation of women/females and sex/gender sensitive reporting (SGR) in clinical cardiovascular trials by region. Summary of (A) share of women/females and (B) participation-prevalence-ratio (PPR) in 1,593 identified trials disaggregated by trial location. Indicated are parity (50%, dashed line), optimal PPR (0.8-1.2, dashed lines) and overall median (dotted line). Sections reporting sex/gender within four categories (demographics, results, endpoint, discussion) of each trial were summarized for all articles of the balanced sample by region (C). Numbers within these bars indicate the share of trials with SGR in at least three of four sections. Because of the reduced number of articles from Sub-Saharan Africa, Northern Africa and Western Asia, Central and Southern Asia, and Oceania in the balanced sample, they were summarized in the category “Other regions” for this analysis. SGR according to the use of sex/gender-sensitive words (“sex”, “gender”, “male”, “female”, “women”, “men”).
